# Rectifying misinformation on the climate intervention potential of ocean afforestation

**DOI:** 10.1038/s41467-024-47134-6

**Published:** 2024-04-09

**Authors:** Victor Smetacek, Mar Fernández-Méndez, Franziska Pausch, Jiajun Wu

**Affiliations:** Alfred Wegener Institute Helmholtz Centre for Polar and Marine Research, Bremerhaven, Germany

**Keywords:** Marine biology, Biooceanography

**arising from** L. T. Bach et al. *Nature Communications* 10.1038/s41467-021-22837-2 (2021)

Open-ocean cultivation of seaweed (macroalgae) biomass, termed ocean afforestation (OAFF), is a nature-based method currently in the planning stage for carbon dioxide removal (CDR) at scales relevant to climate intervention. Bach et al.^1^ cast doubt on its potential efficacy by claiming that “two biogeochemical feedbacks, nutrient reallocation and calcification by encrusting marine life, reduce the CDR efficacy of Sargassum by 20–100%” which has negatively influenced the perception hence evaluation of OAFF compared to the unspecified, technological methods favoured by the authors. In addition to the repudiation of these points by Wang et al.^2^, we show that the green and golden tides^3^ of *Ulva prolifera* and *Sargassum horneri*, respectively, that build up dense mats of free-floating biomass in the millions-of-tonnes range in the eutrophic, central Yellow Sea are more appropriate “natural analogues” for future OAFF than the diffuse Great Atlantic Sargassum Belt (GASB), chosen by Bach et al.^1^. The dense Yellow Sea algal rafts demonstrate that disparate macroalgal species are capable of building up prodigious, free-floating, harvestable biomass at rates depending on the supplies of seeding stocks (from macroalgal thalli fragments) and nutrients (by eutrophication) that can serve as potential crops for seaweed farms based on artificial upwelling of nutrient-rich water in the vast spaces of the open ocean.

The GASB is notorious for seasonally depositing masses of seaweeds on both coasts of the central Atlantic giving the impression of massive biomass production at sea; however, the total biomass produced across an 8850 km stretch of ocean is about 20 million tonnes built up over 6 months^4^, which is orders of magnitude below the gigatonne levels demanded by effective CDR. The latter can only be supported in the open ocean by artificial upwelling of nutrient-rich deep water, supplemented with iron, which Bach et al.^1^ also acknowledge. These “irrigated” seaweed farms of OAFF will be as different from the nutrient-limited GASB, as oases from the surrounding deserts; the carbon-binding efficiency of the algal crops grown in the fields can also be improved over today’s wild forms^5^, to far surpass that of phytoplankton. The C:N:P ratios of macroalgal species are not constant properties but follow gradients driven by the growth stage of the respective thallus, being low immediately after exposure to nutrients when dividing cells constitute a large proportion of the total, but rising with maturation, as cell division rates decline, cell walls thicken, and other grazer protection mechanisms are deployed. It is these grazer protection mechanisms that render macroalgae far superior to phytoplankton for aquafarming and carbon sequestration: (a) none of the benthic grazers of attached macroalgae have followed free-floating macroalgae into the pelagic biome, (b) the mechanical protection offered by robust cell walls confer resistance to breakage by waves pounding exposed coasts and render the thalli rich in structural molecules amenable for use in novel plastics, (c) the robust thalli enable easy harvesting and handling of the crop, (d) the farms can be multipurpose and would take pressure off the current overexploited oceans margins. In any case, the issue of reallocation of artificially upwelled new nutrients does not arise, also not at longer time scales, because OAFF would require <1% of the deep-sea nutrient inventory to sequester the entire stock of fossil fuel CO_2_ released so far.

Large amounts of *Ulva prolifera* and *Sargassum horneri* biomass that grow in a free-floating state in the Yellow Sea are often deposited on adjoining coastlines of China, Korea, and Japan in masses comparable to those of the GASB^6^. Increasing nutrient supply from land runoff allows algal rafts of both species, seeded from fragments shed along the coasts, to grow into dense patches of surface-floating biomass in the course of only 3 weeks^7–10^. In July 2021, more than 450,000 tonnes of drifting seaweed biomass were collected by fishing vessels to reduce damage to aquaculture facilities along the coast^9^. Both the green and golden tides follow dynamics that could be replicated in seaweed farms: seeding algal fragments into nutrient-rich water and subsequent harvesting of the crop at the most appropriate stage for the purposes desired, from algae for human consumption, to raw materials, to CDR. The examples also indicate that macroalgal species other than Atlantic *Sargassum* can function as crops for OAFF and be selected for improving desired traits such as high carbon contents^5^. Thus, the leathery, tough, long (~4 m) fronds of *Sargassum horneri* have C:N ratios of 45^11^. The cosmopolitan green tide genus *Ulva* has lower C/N ratios but 19 has been achieved under cultivation^12,13^, much higher than the Redfield ratio of 6.6 characteristic of phytoplankton and reflected in preformed nutrient ratios in upwelled water. In a successfully run farm, the harvested seaweed could be processed on site to recycle nutrients back to the algal fields, which will improve the carbon uptake efficiency further. The remaining biomass—mostly carbon-rich cell walls—could be converted into various types of structural plastics to make the pipes and the entire infrastructure of the seaweed farms. Verification of CDR would be based on the harvested seaweed biomass and the excess could be deposited in depots on the deep-sea floor for secure storage until required.

Bach et al.^1^ invoke calcification by encrusting epibionts on *Sargassum* as a factor reducing the efficacy of OAFF based on a few high values recorded in the Sargasso Sea. However, healthy seaweeds defend their surfaces against fouling epibionts, but weakened or aged individuals are liable to be infected^14,15^. Under the optimal conditions prevailing in aquafarms, seaweed crops will outgrow the slow-growing, calcifying epibionts, such as the plankton-feeding Bryozoa (the main fouling calcifiers). Besides, the seaweeds will be harvested long before calcifying epibionts can achieve significant coverage. We conclude that farming seaweeds has the potential to become an effective, multipurpose, revenue-generating CDR method^2^. The targeted, interdisciplinary research effort needed to develop the potential of ocean afforestation for large-scale CDR should not be discouraged by the negative conclusions drawn by Bach et al.^1^ from a single, inappropriate analogue. It is equivalent to prejudging the future potential of an evolving giant from the properties of an extant dwarf, stunted by circumstance.
